# Identification of Potential Inhibitors of Histone Deacetylase 6 Through Virtual Screening and Molecular Dynamics Simulation Approach: Implications in Neurodegenerative Diseases

**DOI:** 10.3390/ph17111536

**Published:** 2024-11-15

**Authors:** Anas Shamsi, Moyad Shahwan, Azna Zuberi, Nojood Altwaijry

**Affiliations:** 1 Center for Medical and Bio-Allied Health Sciences Research, Ajman University, Ajman 346, United Arab Emirates; m.shahwan@ajman.ac.ae; 2 College of Pharmacy and Health Sciences, Ajman University, Ajman 346, United Arab Emirates; 3 Division of Reproductive Science in Medicine, Department of Obstetrics & Gynecology, Feinberg School of Medicine, Northwestern University, Chicago, IL 60611, USA; azna.zuberi@northwestern.edu; 4 Department of Biochemistry, College of Science, King Saud University, Riyadh 14511, Saudi Arabia; nojood@ksu.edu.sa

**Keywords:** histone deacetylase 6, neurodegenerative diseases, drug repurposing, small-molecule inhibitors, virtual screening

## Abstract

Background: Histone deacetylase 6 (HDAC6) plays a crucial role in neurological, inflammatory, and other diseases; thus, it has emerged as an important target for therapeutic intervention. To date, there are no FDA-approved HDAC6-targeting drugs, and most pipeline candidates suffer from poor target engagement, inadequate brain penetration, and low tolerability. There are a few HDAC6 clinical candidates for the treatment of mostly non-CNS cancers as their pharmacokinetic liabilities exclude them from targeting HDAC6-implicated neurological diseases, urging development to address these challenges. They also demonstrate off-target toxicity due to limited selectivity, leading to adverse effects in patients. Selective inhibitors have thus been the focus of development over the past decade, though no selective and potent HDAC6 inhibitor has yet been approved. Methods: This study involved an integrated virtual screening against HDAC6 using the DrugBank database to identify repurposed drugs capable of inhibiting HDAC6 activity. The primary assessment involved the determination of the ability of molecules to bind with HDAC6. Subsequently, interaction analyses and 500 ns molecular dynamics (MD) simulations followed by essential dynamics were carried out to study the conformational flexibility and stability of HDAC6 in the presence of the screened molecules, i.e., penfluridol and pimozide. Results: The virtual screening results pinpointed penfluridol and pimozide as potential repurposed drugs against HDAC6 based on their binding efficiency and appropriate drug profiles. The docking results indicate that penfluridol and pimozide share the same binding site as the reference inhibitor with HDAC6. The MD simulation results showed that stable protein–ligand complexes of penfluridol and pimozide with HDAC6 were formed. Additionally, MMPBSA analysis revealed favorable binding free energies for all HDAC6–ligand complexes, confirming the stability of their interactions. Conclusions: The study implies that both penfluridol and pimozide have strong and favorable binding with HDAC6, which supports the idea of repositioning these drugs for the management of neurodegenerative disorders. However, further in-depth studies are needed to explore their efficacy and safety in biological systems.

## 1. Introduction

Histone deacetylases (HDACs) are enzymes essential for transcriptional regulation through the epigenetic modification of histones [1]. Beyond histone modification, some HDAC family members regulate various cellular processes by deacetylating non-histone targets such as α-tubulin, ubiquitin, HSP90, cortactin, peroxiredoxins, and several transcription factors [2]. The HDAC family consists of 18 members that are classified into four classes according to homology with yeast proteins [3]. HDACs that are class I include HDAC1, 2, 3, and 8 because of their similarity to the yeast Rpd3 protein [3]. These enzymes are expressed in all tissues and are involved in the regulation of transcription [4]. Class II HDACs are similar to yeast Hda1, can translocate between cytoplasm and nucleus, and are tissue-specific [5]. HDACs of class II are divided into class IIa (HDAC4, 5, 7, and 9) and class IIb (HDAC6 and 10) [6]. HDAC11 is the only member of class IV and is most similar to the catalytic domains of the class I and II deacetylases [7]. Class I, II, and IV HDACs are referred to as the conventional HDACs and all of them are dependent on Zn^2+^ as a co-factor. Class III HDACs or sirtuins (SIRT1–7) require NAD^+^ and are related to yeast Sir2. HDACs have been implicated in various cellular functions by using isoform-specific knockdown and treatment with HDAC inhibitors [8]. Small-molecule isoform-specific inhibitors are expected to be more effective and less toxic for the long-term treatment of cancer and neurodegenerative diseases [9]. Therefore, much effort has been devoted to elucidating the roles of individual HDAC isoforms, which has been followed by the design of selective HDAC inhibitors.

In particular, HDAC6 has been the subject of much interest because of its distinct features of structure [10]. It has two catalytic N-terminal deacetylating domains, a C-terminal ZnF-UBP domain, and an SE14 tetradecapeptide repeat domain [11]. In addition to these, two leucine-rich nuclear export sequences are present to maintain it in the cytosol. HDAC6 targets non-histone proteins including α-tubulin, poly-ubiquitinated proteins, and HSP90, involved in cell growth, migration, survival, protein degradation, and intracellular transport [12]. These processes are important for postmitotic cells such as neurons because the neurons depend on the proper degradation of proteins and transport of proteins to the axons. Consequently, HDAC6 is being explored as a therapeutic target for cancers and various neurodegenerative diseases such as Alzheimer’s disease (AD), Parkinson’s disease (PD), Huntington’s disease (HD), and amyotrophic lateral sclerosis (ALS) [13,14].

Drug repurposing is an effective approach to the development of new therapies, which can help to overcome the problems associated with high costs and long periods required for the development of new drugs [15]. This approach builds on the safety and pharmacokinetic data of FDA-approved drugs and can offer effective treatments for diseases other than those for which the drugs were initially developed [16]. As with many neurodegenerative diseases, there are few therapeutic options and many of these are not effective; drug repurposing is a promising approach to finding new treatments. The currently available HDAC6-targeting drugs face certain issues, such as poor ability to cross the blood–brain barrier and lack of high specificity [17]. Therefore, through the process of drug repositioning, we hope to find molecules that will efficiently target HDAC6 but will not be associated with the mentioned drawbacks, thus offering new treatment approaches to neurodegenerative diseases.

In this study, we focused on the HDAC6 protein for structure-based drug repurposing. Therefore, we used an integrated screening involving initially the molecular docking technique to identify the most appropriate drug molecules for interaction with the HDAC6 protein. These studies are useful in drug discovery and in drug repositioning as this approach is cheaper and faster than the conventional methods. A set of 3500 FDA-approved drug molecules was obtained from the DrugBank database [18]. The best drug molecules were selected based on the binding affinity and important interactions with HDAC6. The screened molecules were further evaluated for their drug profile and their docked complexes with HDAC6 were further analyzed at the atomic level employing molecular dynamics (MD) simulations followed by essential dynamics approaches.

## 2. Results and Discussion

### 2.1. Molecular Docking Screening

Molecular docking is a widely used computational technique used to predict the preferred orientation and conformation of a ligand within the binding site of a protein receptor [19]. Here, a set of 3500 FDA-approved molecules was obtained from the DrugBank database for docking screening (Supplementary Table S1). The docking screening was carried out using InstaDock to find out the high-affinity binders of HDAC6. The reliability of the docking protocol was validated through redocking of the co-crystallized inhibitor trichostatin A with HDAC6. A comparison of the docking poses with the original co-crystallized pose demonstrated high similarity, confirming the accuracy of the docking method. The alignment of the docked and crystallographic poses underscores the precision of the protocol (Supplementary Figure S1). This validation reinforces the credibility of the docking approach and its potential utility in future molecular docking studies. The docking process entailed scoring all the molecules in the library, where the best 10 molecules were selected based on their docking scores with HDAC6, as shown in Table 1. These scores, which varied from −9.3 to −10.5 kcal/mol, show the strength of the interaction of each ligand with HDAC6. It is observed that the lower the docking scores, the better the binding affinities. All the selected molecules showed better binding affinity than the reference co-crystallized inhibitor trichostatin A, with a docking score of −6.6 kcal/mol (PDB IDs: 5EDU, TSN). In our docking studies, the zinc ion was removed by default in the InstaDock setup. As the zinc ion plays a critical role in the active site of HDAC6, particularly for inhibitors like trichostatin A that chelate zinc via their hydroxamate group, its removal may have contributed to the lower binding affinity observed in our docking calculations.

To further characterize the binding interactions, several additional parameters were analyzed, i.e., pKi ligand efficiency and torsional energy. pKi relates to the binding efficiency between the ligand and the target from the docking scores. A higher pKi value corresponds to a stronger predicted binding affinity. This measure provides insight into the potential effectiveness of the inhibitor. Ligand efficiency is the binding affinity divided by the molecular size in the form of non-hydrogen atoms. It permits the determination of binding affinity differences while at the same time keeping the size of the molecule in consideration since the method distinguishes between potent and efficient ligands in terms of their binding interactions. Torsional energy is the energy related to the rotation of a ligand around its single bond. Lower torsional energy normally points to a lesser amount of stress within the molecule and, hence, the ligand is more conformationally stable once bound to the target. This parameter can affect the binding specificity and stability of the protein–ligand binary complex. All these parameters are, in a way, closer to giving a fuller picture of the binding properties of each molecule. From the outcomes of the docking study, it can be concluded that the screened molecules are quite adequate in terms of their binding energies and properties, which should encourage further exploration and fine-tuning of the identified compounds as potential inhibitors of HDAC6.

### 2.2. PASS Analysis and Drug Profiling

The PASS server was used to predict the biological activity of the molecules that were found through the docking screening process [20]. In this study, PASS analysis was used to assess the biological activity of the molecules, which were ranked at the top of the list. Among the 10 molecules which were selected from the docking screening, two molecules, namely penfluridol and pimozide, were found to be potential candidates in drug profiling for the desired biological activities (Table 2). The PASS analysis was conducted to evaluate the potential bioactivities of penfluridol, pimozide, and trichostatin A. Since these compounds are well-known drugs with established pharmacological profiles, their predicted bioactivities in the PASS analysis are expected and consistent with their known functions. However, PASS also highlights additional potential activities for these compounds beyond their well-established therapeutic roles, providing a broader perspective on their possible pharmacological effects. The PASS analysis showed properties related to acute neurologic disorder treatment, analgesic, antineurotic, antipsychotic, and antimigraine properties, and highlighted that these molecules have a high ability to treat neurodegenerative diseases. It is believed that when the probability of a molecule having the expected biological activity is greater than the probability of being inactive (Pa > Pi), the chance of the molecule being active is high. Among the studied drugs, penfluridol and pimozide had the highest Pa values, which varied from 0.426 to 0.786, which shows that there is a high possibility of treating neurological disorders. The PASS analysis results showed that penfluridol and pimozide are the molecules with the most favorable biological activities in the context of repurposing drugs against HDAC6.

### 2.3. Interaction Analysis

**Figure 1:**
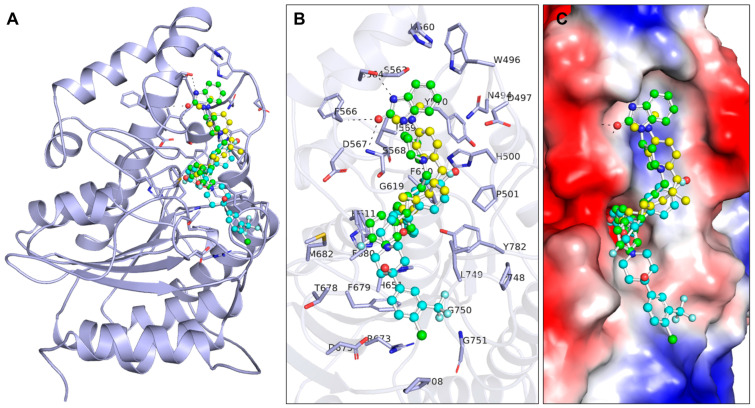
Interaction pattern of selected molecules with HDAC6. (**A**) Binding pattern of HDAC6 with Penfluridol (cyan), Pimozide (green), and Trichostatin A (yellow). (**B**) Magnified view of HDAC6 binding pocket residues interacting with Binding pattern of HDAC6 with Penfluridol, Pimozide, and Trichostatin A. (**C**) Surface potential view of HDAC6 with the selected drug molecules.

In the case of using drugs for new targets, it is crucial to examine the relationships within protein–ligand complexes to achieve the desired effect and avoid unwanted binding [21]. In the present work, the binding modes and interactions of the repurposed drugs penfluridol and pimozide were investigated along with a reference molecule trichostatin A using PyMOL and Discovery Studio Visualizer (Figure 1). These docking simulations produced 27 conformers of these drugs bound to HDAC6 and offered more information about the interaction patterns of the drugs (Figure 1A). Penfluridol and pimozide had several important interactions and the most favorable binding modes in the binding pocket of HDAC6 as in the case of trichostatin A (Figure 1B). Both compounds, penfluridol and pimozide, occupied the active site binding pocket of HDAC6, though the binding pose of penfluridol differed significantly from that of the reference compound, trichostatin A (Figure 1C). These results indicate that penfluridol and pimozide might have a high potential to be exploited as HDAC6 inhibitors with the possibility of further drug development. However, further experimental validation is necessary to determine the specific inhibitory effects and therapeutic potential of penfluridol and pimozide as HDAC6 inhibitors.

**Figure 2:**
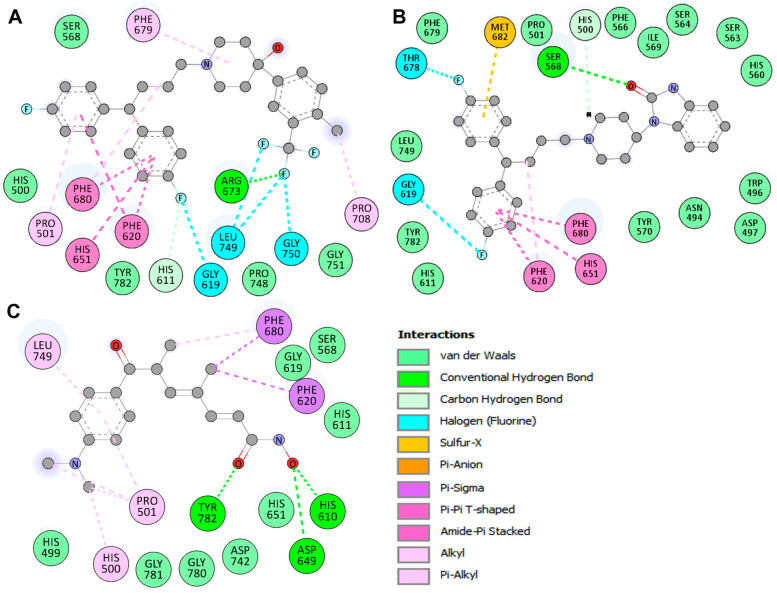
Binding residues of HDAC6 and their interactions with (**A**) Penfluridol, (**B**) Pimozide, and (**C**) Trichostatin A. Hydrogen atoms from the hydroxyl group (OH) are hidden for clarity.

Further, the Discovery Studio Visualizer tool was utilized for detailed interaction analysis (Figure 2). Penfluridol formed several types of interactions with HDAC6 including hydrogen bonds involving residues His611 and Arg673, halogen (fluorine) bonds involving residues Gly619, Leu749, and Gly750, alkyl bonds involving residues Pro501, Phe679, Phe680, and Pro708, pi–pi T-shaped and amide–pi stacked bonds formed by residues Phe620, His651, and Phe680, and van der Waals interactions involving residues His500, Ser568, Pro748, Gly751, and Tyr782 (Figure 2A). At the same time, pimozide had various close interactions with HDAC6 such as hydrogen bonds involving residues His500 and Ser568, halogen (fluorine) bonds involving residues Gly619 and Thr678, pi–anion bonds with residue Met682, alkyl bonds by residue Phe620, pi–pi T-shaped and amide–pi stacked bonds involving residues Phe620, His651, and Phe680, and van der Waals interactions formed by residues Asn494, Trp496, Asp497, Pro501, His560, Ser563, Ser564, Phe566, Ile569, Tyr570, His611, Phe679, Leu749, and Tyr782 (Figure 2B). Similarly, the co-crystallized reference inhibitor trichostatin A had interactions with HDAC6 such as hydrogen bonds with residues His610, Asp649, and Tyr782, pi–sigma bonds created by residues Phe620, and Phe680, alkyl bonds formed by residues His500, Pro501, Phe680, and Leu749, and van der Waals interactions involving residues His499, Ser568, His611, Gly619, His651, Asp742, Gly780, and Gly781 (Figure 2C). Among all interactions, penfluridol formed a hydrogen bond at active site residue His611 while pimozide and trichostatin A formed van der Waals interactions involving residue His611 which is crucial for HDAC6 protein function. These findings recommended that these compounds are explored for further investigation using MD simulations to understand their time-evolution binding mechanism and stability with HDAC6.

### 2.4. MD Simulations

MD simulation is a core and crucial methodology of molecular modeling and computational design for the study of the dynamics and temporal evolution of molecular systems [22]. Here, we investigated four molecular systems, including one free HDAC6 protein and three HDAC6–drug complexes. Before analyzing the deviation, fluctuation, compactness, etc. of the systems, we calculated potential and kinetic energies to ascertain the stability of the systems. The mean potential energies of HDAC6 and HDAC6-Penfluridol, HDAC6-Pimozide, and HDAC6-Trichostatin A complexes were −799,096 kJ/mol, −552,096 kJ/mol, −552,388 kJ/mol, and −551,954 kJ/mol, respectively. The mean kinetic energies of HDAC6 and HDAC6-Penfluridol, HDAC6-Pimozide, and HDAC6-Trichostatin A complexes were 155,797 kJ/mol, 111,168 kJ/mol, 111,173 kJ/mol, and 111,089 kJ/mol, respectively. While the kinetic energy of the HDAC6–drug complexes decreased compared to the free HDAC6 protein, the potential energy became more negative. This increase in the magnitude of the negative potential energy values indicates a more stable and favorable interaction between the protein and the ligands. A more negative potential energy suggests a more stable system, as the drug-bound complexes exhibit stronger interactions that lower the system’s overall energy. Therefore, the observed decrease in kinetic energy, coupled with more negative potential energy, reflects the stabilization of the HDAC6–drug complexes compared to the free HDAC6 protein.

### 2.5. Structural Deviation Analysis

**Figure 3:**
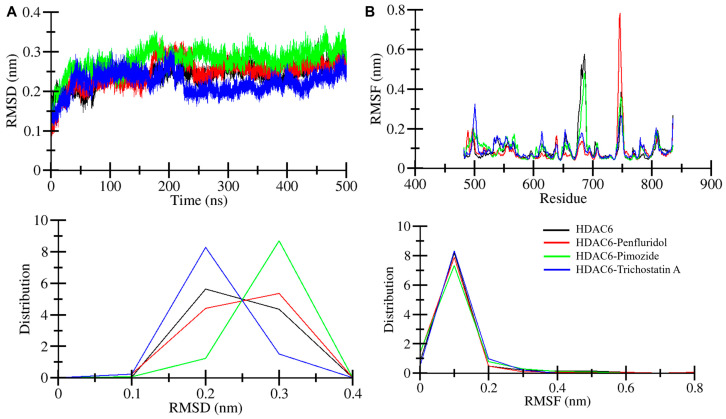
Time-evolution dynamics of HDAC6. (**A**) Structural deviation analysis plot of RMSD of HDAC6, HDAC6-Penfluridol, HDAC6-Pimozide, and HDAC6-Trichostatin A. (**B**) Residual fluctuation analysis plot of RMSF of HDAC6, HDAC6-Penfluridol, HDAC6-Pimozide, and HDAC6-Trichostatin A. The lower panels show the RMSD and RMSF distribution plots of PDFs.

Root mean square deviation (RMSD) is a useful statistical parameter to measure structural deviation [23]. RMSD was employed to monitor the overall stability of the protein–ligand complex during the 500 ns MD simulation. By calculating the RMSD over the entire complex, we can assess whether significant conformational changes occurred and whether the complex remained stable throughout the simulation. Average RMSD values of the systems were calculated to measure how much structural deviation occurred in HDAC6 after drug interactions. Table 3 represents mean RMSD values of HDAC6 and HDAC6-Penfluridol, HDAC6-Pimozide, and HDAC6-Trichostatin A complexes that were 0.24 nm, 0.24 nm, 0.27 nm, and 0.22 nm respectively. HDAC6 and the HDAC6-Penfluridol complex had similar mean RMSDs, the HDAC6-Pimozide complex shows a small increase in RMSD while the HDAC6-Trichostatin A complex indicates a lesser RMSD value. The maximum RMSD value was also calculated to obtain the highest deviation point of the systems (Figure 3A). HDAC6 and the HDAC6-Penfluridol complex show similar maximum RMSDs of 0.33 nm. The HDAC6-Pimozide complex’s maximum RMSD was 0.36 nm which is a bit higher while the HDAC6-Trichostatin A complex had a 0.31 nm maximum RMSD throughout the simulation. As shown in the main RMSD plot and distribution plot, the HDAC6-Pimozide complex indicated by green has a slightly higher RMSD throughout the simulation (Figure 3A, upper panel). The RMSD distribution in the probability distribution function plot (PDF) is mostly from 0.2–0.3 nm (Figure 3A, lower panel). However, the overall deviation result indicates all three complex systems increase in stability throughout the 500 ns long simulation.

Root mean square fluctuation (RMSF) is another useful parameter to calculate residual fluctuation in simulated molecular systems. RMSF was employed to understand the flexibility of specific residues in the protein. Higher RMSF values indicate regions of the protein that exhibit greater fluctuations, often corresponding to loop regions or flexible domains. These fluctuations can reveal areas that undergo conformational adjustments during ligand binding, providing insights into the dynamic behavior of the protein. The average RMSF value was calculated to ascertain overall residual fluctuations in the systems. The average values of HDAC6 and HDAC6-Penfluridol, HDAC6-Pimozide, and HDAC6-Trichostatin A complexes were 0.09 nm, 0.08 nm, 0.10 nm, and 0.09 nm, respectively (Table 3). Regions of high RMSF indicate areas of greater flexibility, for example, residues from 740–750 of the HDAC6-Penfluridol complex have the highest fluctuation, which is 0.78 nm (Figure 3B). The maximum fluctuations of HDAC6, HDAC6-Pimozide, and HDAC6-Trichostatin A were 0.57 nm, 0.48 nm, and 0.32 nm, respectively (Figure 3B, upper panel). This flexibility suggests local conformational changes that could facilitate ligand binding or stabilize the interaction. In contrast, residues in the HDAC6-Trichostatin A complex showed lower RMSF values (~0.32 nm), indicating more rigidity, which correlates with the stable binding observed in the complex. The overall result as an RMSF distribution plot is presented in Figure 3B, and the lower panel shows minimal fluctuations with similar patterns during the simulation suggesting structural stability.

### 2.6. Compactness Analysis

**Figure 4:**
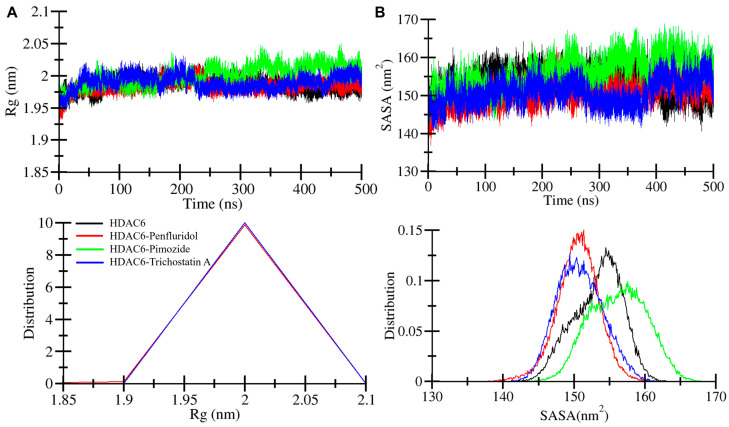
Structural compactness assessment plot of (**A**) *R*g of HDAC6, HDAC6-Penfluridol, HDAC6-Pimozide, and HDAC6-Trichostatin A. (**B**) Solvent-accessible surface area assessment plot of HDAC6, HDAC6-Penfluridol, HDAC6-Pimozide, and HDAC6-Trichostatin A. The lower panels show *R*g and SASA distribution plots of PDFs. Black, red, green, and blue represent HDAC6, HDAC6-Penfluridol, HDAC6-Pimozide, and HDAC6-Trichostatin A, respectively.

The compactness of the protein–drug complexes was investigated by calculation of the radius of gyration (*R*g) [24]. This parameter is crucial to understanding whether the protein undergoes significant unfolding or structural expansion during the simulation. A stable *R*g value, as observed in our simulations, indicates that the protein did not undergo major unfolding, maintaining a stable structure when bound to the ligands. This is significant in ensuring that the binding of the ligands does not disrupt the overall folding of the protein. The result presented in Figure 4 indicates the time evolution and complex stability and compactness. The HDAC6-Pimozide complex in green shows minimally increasing *R*g after 250 ns of simulation. The other two complexes, HDAC6-Penfluridol and HDAC6-Trichostatin A, in red and blue, respectively, almost overlap with the free HDAC6 protein (Figure 4A). Average *R*g values of HDAC6 and HDAC6-Penfluridol, HDAC6-Pimozide, and HDAC6-Trichostatin A complexes were 1.98 nm, 1.98 nm, 2.00 nm, and 1.98 nm, respectively (Table 3). The maximum *R*g values of HDAC6 and HDAC6-Penfluridol, HDAC6-Pimozide, and HDAC6-Trichostatin A complexes were 2.02 nm, 2.02 nm, 2.04 nm, and 2.03 nm, respectively (Figure 4A, upper panel). These statistical findings along with the main *R*g and distribution plot suggested the complexes were stable throughout the entire simulations (Figure 4A, lower panel).

Solvent-accessible surface area (SASA) is a part of the protein’s surface area which can interact with the surrounding solvent [25]. It is used to measure the folding and unfolding pattern of biological systems. This is a useful parameter for monitoring folding and unfolding events, as changes in SASA often correlate with changes in the protein’s exposure to the solvent. Here we calculated the average SASA values of the HDAC6 and HDAC6-Penfluridol, HDAC6-Pimozide, and HDAC6-Trichostatin A complexes which were 153.3 nm^2^, 150.5 nm^2^, 156.2 nm^2^, and 150.8 nm^2^, respectively (Table 3). The HDAC6-Pimozide complex is the only system that shows a larger surface area accessible to a solvent which may be due to the binding adjustment of the ligand. However, it does not show any worse impact on the protein folding as presented by the SASA plot (Figure 4B). Maximum SASA values of the HDAC6 and HDAC6-Penfluridol, HDAC6-Pimozide, and HDAC6-Trichostatin A complexes were found to be 164.9 nm^2^, 162.8 nm^2^, 168.8 nm^2^, and 163.1 nm^2^, respectively (Figure 4B, upper panel). The calculated values, main SASA, and distribution plot indicate that the HDAC6-Pimozide complex had a minimally increasing pattern after 200 ns of simulation, but no major drift changes occurred till the end of the simulation (Figure 4B, lower panel). Overall, SASA findings correlate with *R*g parameters, suggesting complex stability and compactness.

### 2.7. Hydrogen Bond Analysis

**Figure 5:**
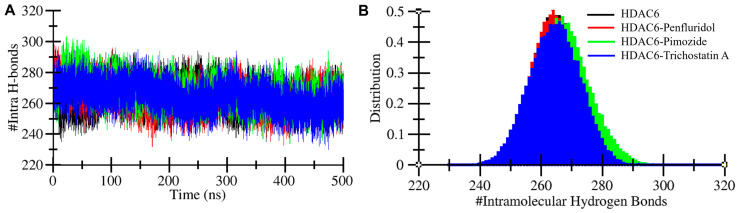
Intramolecular hydrogen bonding. (**A**) Intramolecular hydrogen bond plot of HDAC6 protein before and after interactions of Penfluridol, Pimozide, and Trichostatin A. (**B**) Distribution plot of intramolecular hydrogen bonds.

In biological macromolecules, hydrogen bonds play a vital role in maintaining three-dimensional conformational shape and stability [26]. To examine the stability of HDAC6 before and after interactions with the elucidated molecules, we calculated intramolecular hydrogen bonds. The average numbers of bonds formed within the protein were 264, 264, 267, and 264 for HDAC6 and HDAC6-Penfluridol, HDAC6-Pimozide, and HDAC6-Trichostatin A complexes, respectively (Table 3). The maximum numbers of intramolecular bonds of HDAC6 and HDAC6-Penfluridol, HDAC6-Pimozide, and HDAC6-Trichostatin A complexes were 297, 300, 303, and 295, respectively (Figure 5). Overall, the calculated result revealed the complexes made a higher number of bonds than proteins in free form. The plot depicted in Figure 5A indicates hydrogen bond dynamics with 500 ns time evolution. The distribution plot also indicates a higher number of bonds formed by the HDAC6-Pimozide complex represented by green (Figure 5A).

**Figure 6:**
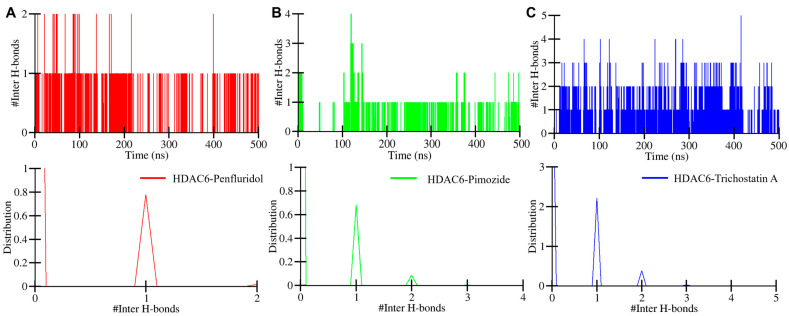
Intermolecular hydrogen plot of HDAC6–drug complexes. (**A**) HDAC6-Penfluridol, (**B**) HDAC6-Pimozide, and (**C**) HDAC6-Trichostatin A.

Additionally, the intermolecular bond formed between protein and drug molecules was also assessed to elucidate complex stability [27]. To investigate the stability and dynamics of protein–ligand interactions, we analyzed the contacts between HDAC6 and the ligands (penfluridol, pimozide, and trichostatin A) throughout the 500 ns molecular dynamics simulation. The analysis focused on hydrogen bonds formed intermolecularly within the protein–ligand complexes. Figure 6 represents the maximum hydrogen bonds formed within HDAC6-Penfluridol, HDAC6-Pimozide, and HDAC6-Trichostatin A complexes which were two, four, and five, respectively. The analysis revealed that all three complexes maintained stable interactions with key residues in the HDAC6 binding pocket. The number of hydrogen bonds fluctuated slightly but remained consistent overall, with penfluridol maintaining an average of 1–2 hydrogen bonds, pimozide showing 1–2 hydrogen bonds, and trichostatin A exhibiting 2–3 hydrogen bonds throughout the simulation. A distribution plot of all three complexes showing bonding time throughout the simulation was made. One hydrogen bond in all three complexes has a higher distribution than other bonds which formed between the protein and drug for a shorter time span (Figure 6, lower panels). This consistency in contacts over the simulation suggests that the ligands remained stably bound to HDAC6, supporting their potential as repurposed inhibitors.

### 2.8. Secondary Structure Alteration Analysis

**Figure 7:**
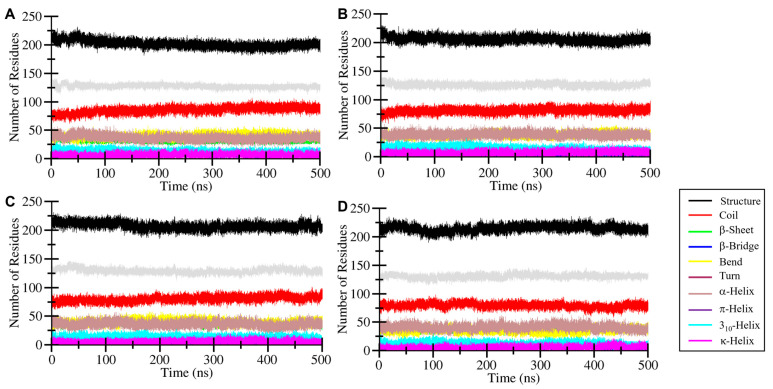
Secondary structure elements representation of (**A**) HDAC6, (**B**) HDAC6-Penfluridol, (**C**) HDAC6-Pimozide, and (**D**) HDAC6-Trichostatin A.

Secondary structure analysis includes changes in the different elements of the protein’s secondary structure before and after the interaction of drug molecules was examined [28]. The secondary structure analysis was performed to monitor changes in the protein’s secondary structure elements, such as α-helices and β-sheets, during the simulation. This is important because significant alterations in secondary structure can indicate destabilization of the protein fold upon ligand binding. The DSSP algorithm was utilized to plot different elements of the HDAC6 protein before and after the interaction of the drug molecules. Figure 7 indicates elements of protein in different colors, such as black for structures showing increased residues over time after the interaction of drugs. HDAC6-Penfluridol, HDAC6-Pimozide, and HDAC6-Trichostatin A complexes show considerable increases in the β-sheets. The HDAC6-Pimozide and HDAC6-Trichostatin A complexes also increased in α-helixes while the HDAC6-Pimozide complex residues were similar to those of the free protein (Table 4). Here, we observed no major changes in the secondary structure of HDAC6, suggesting that ligand binding does not disrupt the structural integrity of the protein. Minor reductions in participating residues of coils and β-bridges were observed due to the binding adjustment of drugs. Overall, in secondary structure analysis, no major alteration was predicted during the entire simulation period which shows that all complexes were stable.

### 2.9. Principal Component Analysis

**Figure 8:**
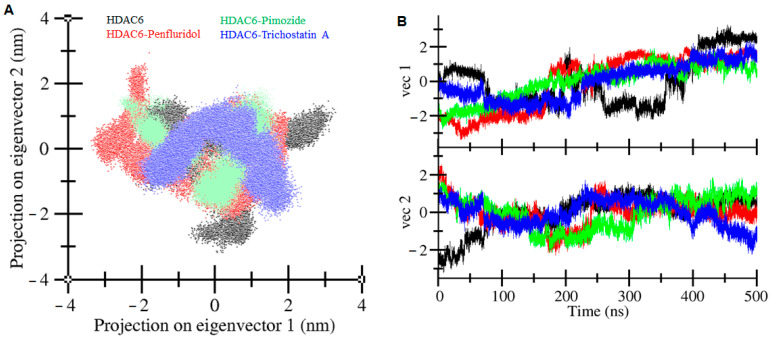
Principal component analysis. (**A**) A 2D projection plot of HDAC6 protein and its complexes with Penfluridol, Pimozide, and Trichostatin A, indicated by different colors. (**B**) Time-dependent eigenvector representation of HDAC6 protein and its complexes with Penfluridol, Pimozide, and Trichostatin A, indicated by different colors.

The principal component analysis (PCA) was performed to identify the primary modes of correlated motion within HDAC6 and its complexes with the selected drug molecules. This approach provides insights into HDAC6′s conformational dynamics and stability under different binding conditions, focusing on large-scale motions that are often associated with functional behavior in proteins. PCA was performed using the 500 ns trajectories of Cα atoms from the MD simulations of HDAC6 in complex with penfluridol, pimozide, and the reference compound, trichostatin A (Figure 8). These trajectories were analyzed to capture the essential dynamics and correlated motions of the protein–drug complexes. The first two principal components (PC1 and PC2), which captured the largest variance in the system’s atomic coordinates, were used to interpret the primary motions induced by ligand binding. For HDAC6 in the free form, the eigenvector values spanned from −2.28 nm to 3.28 nm for PC1 and −3.19 nm to 1.65 nm for PC2. Upon binding with penfluridol, pimozide, and trichostatin A, the eigenvector values changed to −3.39 nm to 2.01 nm, −2.76 nm to 1.90 nm, and −2.18 nm to 2.37 nm on PC1 and from −2.25 nm to 2.93 nm, −2.11 nm to 1.89 nm, and −2.22 nm to 1.92 nm on PC2, respectively (Figure 8A). These shifts reflect variations in the conformational flexibility and stability of HDAC6 in each complex.

The HDAC6-Penfluridol complex showed a slightly larger spread along PC2, suggesting more motion of certain loop regions in accordance with the higher flexibility observed in RMSF analysis. This flexibility may facilitate penfluridol to move within the binding pocket and hence increase its inhibitory potency. The conformational regions that the HDAC6-Trichostatin A complex occupied were smaller than the control, indicating that this binding mode was stable and rigid, which may be related to its high binding constant. The time-dependent eigenvector plot also reveals more dynamic changes in HDAC6 during the simulation time and the relatively stable vector 2 in all the complexes after the initial stabilization phase of 100 ns (Figure 8B). Altogether, the PCA and RMSF data suggest that all three ligands stabilize the binding conformations in HDAC6, though penfluridol has a little more flexibility around the loop regions of the protein. This added flexibility may contribute to its favorable binding profile and potential as an HDAC6 inhibitor.

### 2.10. Free Energy Landscapes

**Figure 9:**
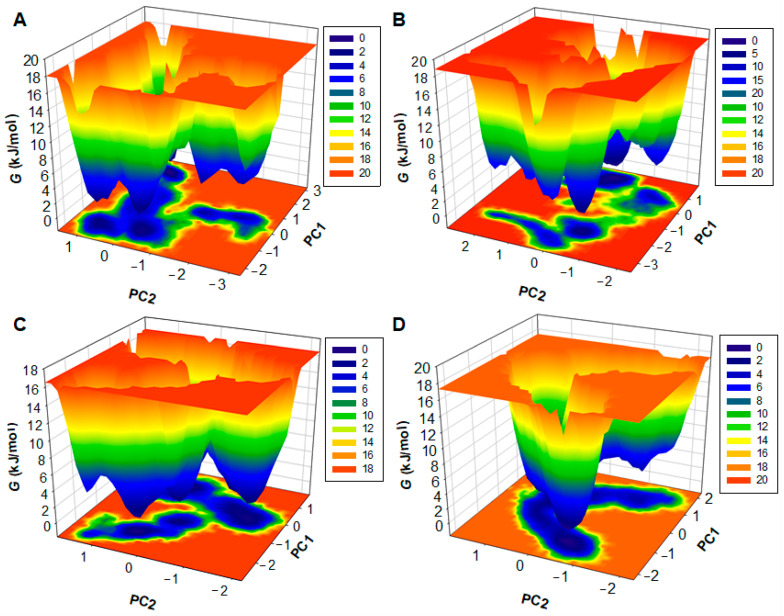
Three-dimensional Gibbs free energy maps of (**A**) HDAC6, (**B**) HDAC6-Penfluridol, (**C**) HDAC6-Pimozide, and (**D**) HDAC6-Trichostatin A.

The FELs were generated to understand the energy distribution of the protein-folding pathway during molecular dynamics. Free energy contour maps of protein and complexes were generated to measure energy distribution which help to elucidate stability and folding mechanism. Different colors in contour maps, red, yellow, orange, and blue, were generated to analyze the energy range from maximum to minimum. The energy ranges of HDAC6 and HDAC6-Penfluridol, HDAC6-Pimozide, and HDAC6-Trichostatin A complexes were 0 to 18 kJ/mol, 0 to 18.8 kJ/mol, 0 to 16.5 kJ/mol, and 0 to 17.3 kJ/mol, respectively (Figure 9). The dark blue basin in the contour maps indicates minimum energy or energy close to zero which suggests a stable state of the protein. Figure 9A of the HDAC6 map shows four dark blue energy basins which indicate different meta-states. The HDAC6-Penfluridol complex has one large and two small blue basins, the HDAC6-Pimozide complex map has three large dark blue basins with one small basin (Figure 9B,C). The HDAC6-Trichostatin A complex has one combined large blue basin which has three small dark blue basins (Figure 9D). The height of the HDAC6-Penfluridol contour map was lower than other maps. Overall, FEL projections along with principal component plots revealed that complexes achieved their global minima and thus showed considerable stability. Taken together, the study indicates that penfluridol and pimozide have promising binding potential with stability with HDAC6 and have appropriate drug profiles to be exploited as repurposed drugs in neurodegenerative diseases.

### 2.11. MMPBSA Analysis

MMPBSA analysis was conducted using the gmx_MMPBSA module in GROMACS to estimate the binding free energy of the HDAC6–ligand complexes. This thermodynamic parameter reflects the energy change associated with the formation of the protein–ligand complex, providing insight into the strength of their interactions [29]. The analysis yielded binding free energy components, including van der Waals forces and electrostatic contributions (Table 5). The results demonstrate that all HDAC6–ligand complexes exhibit favorable binding free energies, indicating stable interactions. Among the complexes, HDAC6-Penfluridol displayed the highest binding affinity, suggesting it forms the most stable complex.

## 3. Material and Methods

### 3.1. Molecular Docking Screening Protocol

Primarily for the structure-guided virtual screening, a molecular docking-based strategy was used to screen molecules with high binding affinity to HDAC6. The process involved the use of MGL AutoDock tools [30], InstaDock 1.2 [31], PyMOL 3.1 [32], and Discovery Studio Visualizer 2023 [33], along with various web-based applications. Three-dimensional structural coordinates of HDAC6 were obtained from the Protein Data Bank (accession number: 5EDU, chain B) and optimized for the docking studies with the help of InstaDock v1.2 and AutoDock tools. The structural preprocessing includes rebuilding missing residues, adding hydrogen to polar atoms, and assigning correct atom types. The drug library was obtained from the DrugBank database and prepared for virtual screening using InstaDock v1.2. The docking simulations were carried out in a blind search space with a grid with dimensions of 65 Å, 77 Å, and 63 Å, with the respective coordinates at the center of 15.135 Å, −33.815 Å, and 92.124 Å for the X, Y, and Z axes, respectively. The spacing of the grid was set to 1 Å where all the heavy atoms of the protein structure were included in the search space. This search space was large enough to accommodate whole protein where all the ligands were free to move and search for their favorable binding sites. After the docking study, all the compounds were ranked based on their binding affinity, and the best compounds were selected for further analysis.

### 3.2. Biological Capability and Interaction Study

The biological activity of the screened molecules was determined by using the PASS web server, which compares the structure of a molecule to a training set of biological activities [20]. PASS calculates the biological activities of the molecules based on the structure–activity relationship and gives the results in the form of ‘probability to be active’ (Pa) and ‘probability to be inactive’ (Pi). A higher value of Pa indicates a higher possibility of the molecule exhibiting the predicted biological property. After the PASS analysis, the interactions and binding models of the screened molecules were studied. Interactions between the selected molecules and HDAC6 were analyzed using the PyMOL 3.1 tool. Furthermore, Discovery Studio Visualizer was used to perform a detailed analysis of the interactions that might occur in the binding pocket of HDAC6. Molecules that showed binding with the key residues, particularly the active site residues like His611, were considered for further analysis.

### 3.3. MD Simulations Protocol

All-atom MD simulations were performed to study the time evolution of binding dynamics of the HDAC6–drug complexes at the atomic level [34]. The simulations were performed at pH 7.0 using the CHARMM36-jul2022 force field [35] with the TIP3P water model [36] in the GROMACS package [37]. A total of 12,580 solvent molecules were added to the system for solvation. Topology files for the screened molecules (penfluridol, pimozide, trichostatin A) were prepared using the CGenFF web server [38]. The systems were solvated in a cubic box with a minimum distance of 10 Å from the protein to the edge of the box (with dimensions 8.41 × 8.41 × 8.41 nm for x, y, and z, respectively), and 8 Na^+^ were added as counterions to balance the charges on the system. Energy minimization was performed by the steepest descent method for 5000 steps and with a cut-off of 10.0 kJ/mol to minimize steric hindrance. Two equilibration phases were conducted: NVT and NPT ensembles for 1000 ps each, where the parameters that are kept constant are the number of particles, volume, and temperature and number of particles, pressure, and temperature, respectively. The temperature was maintained at 300 K using the Berendsen thermostat with a coupling constant of 0.1 ps during the NVT phase, while pressure coupling during the NPT phase was performed using the Parrinello–Rahman barostat with a pressure of 1 bar and a coupling constant of 2.0 ps. For the long-range electrostatics, the particle mesh Ewald (PME) method was employed, with the Coulomb interactions being calculated within a cut-off radius of 12 Å. A van der Waals cut-off of 1.0 nm was applied. The leapfrog integration algorithm was used with a time step of 2 fs, and all bonds involving hydrogen atoms were constrained using the LINCS algorithm. Temperature coupling was done using the Berendsen algorithm, while the pressure coupling during the NPT equilibration used the Parrinello–Rahman method. The last production run was for 500 ns, and the log files and energy data were collected every 10 ps. The obtained trajectories were further processed and analyzed using GROMACS built-in tools. For the secondary structure analysis, the DSSP algorithm was used [28].

### 3.4. Principal Component Analysis Protocol

Principal component analysis (PCA) was performed to capture the essential dynamics of HDAC6 and its complexes, providing insights into the primary modes of motion that influence protein stability and flexibility upon ligand binding. This technique reduces the dimensionality of the data, focusing on significantly correlated motions relevant to functional conformational changes [39]. The covariance matrix was constructed from a concatenated set of atomic trajectories representing all simulated states of HDAC6, including the APO form and ligand-bound complexes. This approach enables a holistic capture of the protein’s structural behavior across varying states, facilitating a robust analysis of dynamic responses induced by each ligand. The covariance matrix captures the variance and covariance between pairs of atomic coordinates and is defined as:

*C_ij_* = ⟨(*x_i_* − ⟨*x_i_*⟩) (*x_j_* − ⟨*x_j_*⟩)⟩

where *x_i_* and *x_j_* are the coordinates of the *i*th and *j*th atoms of the systems and ⟨*x_i_*⟩ and ⟨*x_j_*⟩ represent the average coordinates of the *i*th and *j*th atoms over the ensemble. Then, the principal components (PCs) are calculated by diagonalization of the covariance matrix.

The analysis concentrated on the first two principal components (PCs), which account for the largest proportion of variance in atomic movements. These components allowed us to identify key structural changes induced by ligand binding, particularly in flexible regions such as loop segments near the binding site.

### 3.5. Free Energy Landscape Generation

The free energy landscape (FEL) provides insights into the stability and conformational changes of biomolecules, typically proteins and protein–ligand systems [40]. FEL analysis helps identify stable states and understand the transitions between them. The FEL can be determined using the following approach:

Δ*G* (*X*) = − *k_B_T lnP*(*X*)

where Δ*G* (*X*) denotes free energy, *k_B_*, *T*, and *P*(*X*) are the Boltzmann constant, absolute temperature, and the probability distribution of the conformation ensemble along the PCs.

### 3.6. MMPBSA Calculation

Molecular mechanics/Poisson–Boltzmann surface area (MMPBSA) is a commonly employed method for estimating the binding free energy of protein–ligand complexes [41]. For this analysis, a short 10 ns segment (from 250 to 260 ns) was extracted from the stable regions of the HDAC6-Penfluridol, HDAC6-Pimozide, and HDAC6-Trichostatin A (control) simulations. Using the gmx_MMPBSA package, binding energy components were calculated. The total binding energy of the complex was determined by the following equation:

Δ*G*_Binding_ = *G*_Complex_ − (*G*_Protein_ + *G*_Ligand_)

where, *G*_Complex_ represents the total free energy of the binding complex, and *G*_Protein_ and *G*_Ligand_ refer to the total free energies of HDAC6 and the compounds penfluridol, pimozide, and trichostatin A, respectively.

## 4. Conclusions

The identification of potent and effective HDAC6 inhibitors is significant for the development of novel therapeutic approaches in neurodegenerative diseases. Here, we employed an integrated in silico strategy to screen a panel of 3500 FDA-approved drugs for their ability to modulate HDAC6. Out of all the compounds screened, penfluridol and pimozide stand out as the most promising drugs for repurposing because of their high binding potential, drug profiles, and stable interactions with HDAC6. Further 500 ns MD simulations supported the stability of protein–ligand complexes through structural deviation, residue fluctuation, compactness, hydrogen bond analysis, PCA, and FEL analysis. The results imply that both penfluridol and pimozide have strong and favorable binding with HDAC6, which supports the idea of repositioning these drugs for the management of neurodegenerative disorders. Additionally, MMPBSA analysis revealed favorable binding free energies for all HDAC6–ligand complexes, confirming the stability of their interactions. However, since this is an in silico analysis, further experimental validation is necessary to determine the biological effectiveness and toxicity of these compounds in vivo. Furthermore, the simulations helped determine the stability of the complexes; however, they cannot capture all the biological factors and side effects. In conclusion, the present study offers a solid foundation for subsequent experimental and clinical research and opens new treatment prospects by using these repurposed drugs to target HDAC6.

## Figures and Tables

**Table 1:** List of screened hits against HDAC6 and their docking parameters.

S. No.	Drug	Binding Affinity (kcal/mol)	pKi	Ligand Efficiency (kcal/mol/non-H atom)	Torsional Energy
1.	Dutasteride	−10.5	7.7	0.2838	1.2452
2.	Bisdequalinium Chloride	−10.2	7.48	0.2318	0
3.	Conivaptan	−10.0	7.33	0.2632	1.2452
4.	Penfluridol	−10.0	7.33	0.2778	2.8017
5.	Pimozide	−9.8	7.19	0.2882	2.1791
6.	Rolapitant	−9.7	7.11	0.2771	2.1791
7.	Lumacaftor	−9.4	6.89	0.2848	1.8678
8.	Difenoxin	−9.4	6.89	0.2938	2.4904
9.	Phenindamine	−9.3	6.82	0.465	0.3113
10.	Acrivastine	−9.3	6.82	0.3577	2.1791
11.	Trichostatin A	−6.6	4.84	0.3	2.1791

**Table 2:** PASS properties of the elucidated molecules with their predicted activity.

S. No.	Drug	Pa	Pi	Activity
1.	Penfluridol	0.786	0.010	Acute neurologic disorder treatment
		0.748	0.007	Analgesic
		0.586	0.005	Antineurogenic pain
		0.662	0.094	Phobic disorder treatment
		0.576	0.080	Antineurotic
2.	Pimozide	0.610	0.011	Antipsychotic
		0.534	0.064	Acute neurologic disorder treatment
		0.475	0.011	Antineurogenic pain
		0.462	0.005	Antimigraine
		0.426	0.056	Neurodegenerative disease treatment
3.	Trichostatin A	0.765	0.002	Histone deacetylase inhibitor
		0.738	0.002	Histone deacetylase 6 inhibitor
		0.734	0.002	Histone deacetylase class IIb inhibitor
		0.693	0.002	Histone deacetylase class II inhibitor
		0.533	0.035	Apoptosis agonist

**Table 3:** Average values calculated post 500 ns MD simulations of HDAC6 protein and HDAC6–drug complexes.

S. No.	Systems	RMSD (nm)	RMSF (nm)	*R*g (nm)	SASA (nm^2^)	Intramolecular H-Bonds
1.	HDAC6	0.24 (±0.03)	0.09 (±0.08)	1.98 (±0.01)	153.3 (±3.42)	264 (±8)
2.	HDAC6-Penfluridol	0.24 (±0.03)	0.08 (±0.08)	1.98 (±0.01)	150.5 (±2.98)	264 (±8)
3.	HDAC6-Pimozide	0.27 (±0.03)	0.10 (±0.07)	2.00 (±0.01)	156.2 (±3.98)	267 (±9)
4.	HDAC6-Trichostatin A	0.22 (±0.03)	0.09 (±0.05)	1.98 (±0.01)	150.8 (±3.26)	264 (±8)

**Table 4:** The average number of participating residues in secondary structure elements in HDAC6 during 500 ns MD simulations. Structure = α-Helix + β-sheet + β-Bridge + Turn.

Systems	Structure	Coil	β-Sheet	β-Bridge	Bend	Turn	α-Helix	Pi-Helix	3_10_-Helix	PPII-Helix
HDAC6	202 (±6)	86 (±6)	31 (±2)	6 (±2)	41 (±4)	37 (±5)	128 (±3)	5 (±0)	14 (±4)	7 (±3)
HDAC6-Penfluridol	206 (±5)	81 (±5)	37 (±1)	4 (±2)	37 (±4)	39 (±4)	126 (±4)	5 (±0)	15 (±4)	8 (±4)
HDAC6-Pimozide	208 (±6)	81 (±5)	37 (±2)	5 (±2)	41 (±4)	37 (±5)	129 (±4)	5 (±0)	14 (±4)	6 (±3)
HDAC6-Trichostatin A	215 (±6)	80 (±5)	37 (±2)	5 (±1)	35 (±4)	42 (±5)	131 (±4)	5 (±0)	13 (±4)	6 (±3)

**Table 5:** MMPBSA calculations of binding free energy for HDAC6–ligand complexes. All the values are in kcal/mol.

Complex	∆E_vdW_	∆E_ele_	∆*G*_gas_	∆*G*_polar_	∆*G*_nonpolar_	∆*G*_sol_	∆*G*_bind_
HDAC6-Penfluridol	−58.60	−5.86	−48.58	20.66	−6.02	12.08	−52.82
HDAC6-Pimozide	−40.12	−6.52	−50.27	18.52	−4.52	12.82	−56.03
HDAC6-Trichostatin A	−46.18	−5.90	−46.50	10.54	−4.08	6.76	−41.70

## Data Availability

Data included in article/Supplementary Materials/referenced in article.

## Supplementary Materials

The following supporting information can be downloaded at: https://www.mdpi.com/article/10.3390/ph17111536/s1, Figure S1: The docked conformation of Trichostatin A with HDAC6 superimposed with its co-crystallized position. The figure was generated using PyMOL, based on the Protein Data Bank coordinates (PDB ID: 5EDU); Table S1: FDA-approved Molecules from DrugBank Database for Docking Screening.
