# Supplementary material for: Identification of Potential Inhibitors of Histone Deacetylase 6 Through Virtual Screening and Molecular Dynamics Simulation Approach: Implications in Neurodegenerative Diseases
Source: Pharmaceuticals (Basel). 2024 Nov 15;17(11):1536. doi: 10.3390/ph17111536 (PMC11597257; doi:10.3390/ph17111536)
Supplement: Supplementary file 1 [file pharmaceuticals-17-01536-s001.zip › Supplementary Figure S1.pptx]

## Slide 1
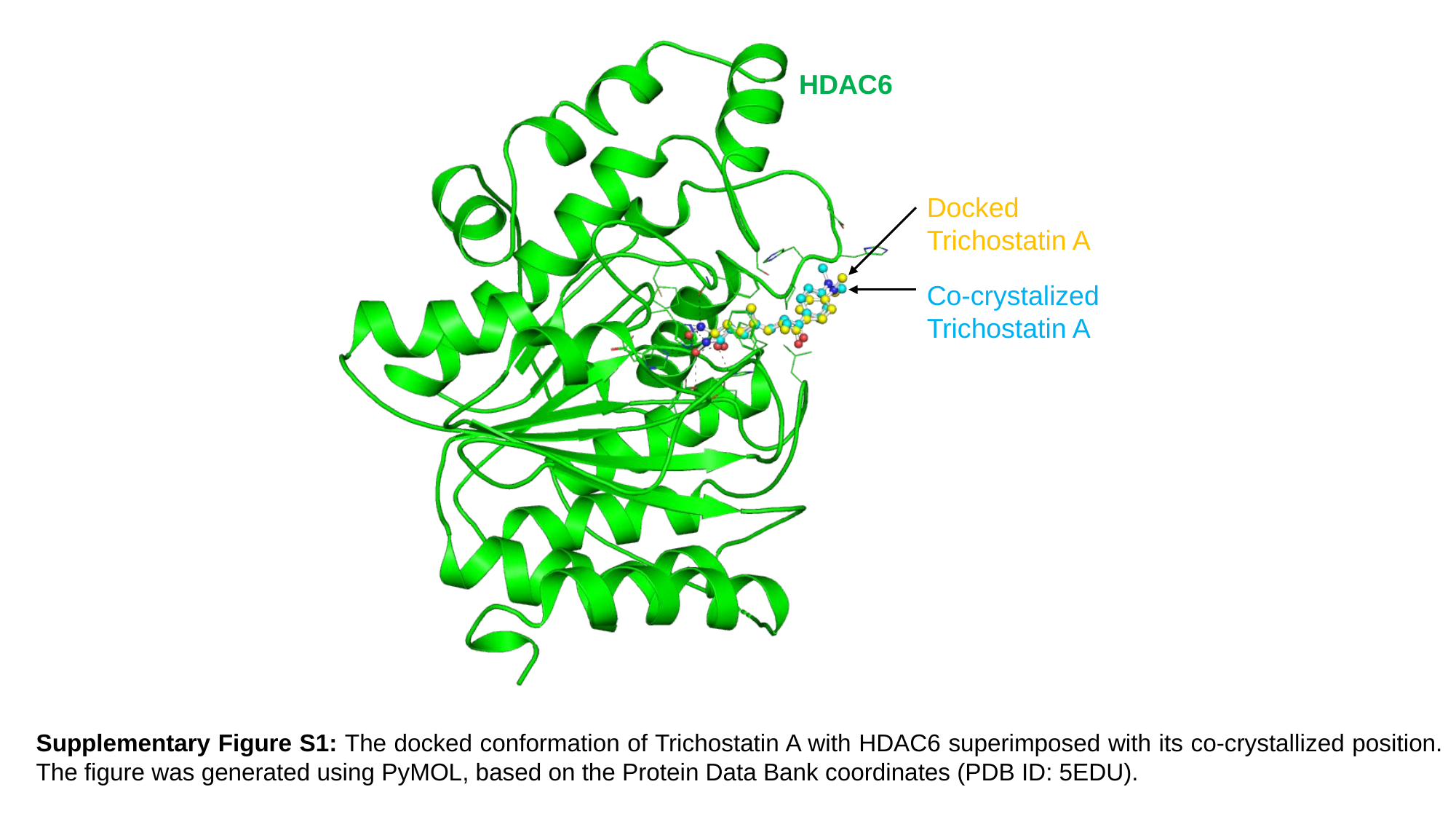

HDAC6
Docked
Trichostatin A
Co-crystalized Trichostatin A
Supplementary Figure S1: The docked conformation of Trichostatin A with HDAC6 superimposed with its co-crystallized position. The figure was generated using PyMOL, based on the Protein Data Bank coordinates (PDB ID: 5EDU).
